# Insight Into Biological Targets and Molecular Mechanisms in the Treatment of Arsenic-Related Dermatitis With Vitamin A *via* Integrated *in silico* Approach

**DOI:** 10.3389/fnut.2022.847320

**Published:** 2022-05-23

**Authors:** Qiuhai Qin, Lixiu Qin, Ruitang Xie, Shuihua Peng, Chao Guo, Bin Yang

**Affiliations:** ^1^Department of Surgery, The People’s Hospital of Gangbei District, Guigang, China; ^2^College of Pharmacy, Guangxi Medical University, Nanning, China; ^3^Department of Pharmacy, Guigang City People’s Hospital, The Eighth Affiliated Hospital of Guangxi Medical University, Guigang, China

**Keywords:** dermatitis, arsenic, drug repurposing, network pharmacology, molecular mechanism

## Abstract

Exposure to arsenic (As), an inorganic poison, may lead to skin lesions, including dermatitis. Vitamin A (VA), a fat-soluble vitamin essential for mucous membrane integrity, plays a key role in skin protection. Although the beneficial actions of VA are known, the anti-As-related dermatitis effects of VA action remain unclear. Hence, in this study, we aimed to interpret and identify the core target genes and therapeutic mechanisms of VA action in the treatment of As-related dermatitis through integrated *in silico* approaches of network pharmacology and molecular docking. We integrated the key VA-biological target-signaling pathway-As-related dermatitis networks for identifying core drug targets and interaction pathways associated with VA action. The network pharmacology data indicated that VA may possess potential activity for treating As-related dermatitis through the effective regulation of core target genes. An enrichment analysis in biological processes further revealed multiple immunoregulation-associated functions, including interferon-gamma production and negative regulation of T-cell activation and production of molecular mediator of immune response. An enrichment analysis in molecular pathways mainly uncovered multiple biological signaling, including natural killer cell mediated cytotoxicity, autophagy, apoptosis, necroptosis, platelet activation involved in cell fate, and immunity regulations. Molecular docking study was used to identify docked well core target proteins with VA, including Jun, tumor protein p53 (TP53), mitogen-activated protein kinase-3 (MAPK3), MAPK1, and MAPK14. In conclusion, the potential use of VA may suppress the inflammatory stress and enhance the immunity against As-related dermatitis. In the future, VA might be useful in the treatment of dermatitis associated with As through multi-targets and multi-pathways in clinical practice.

## Introduction

Pollution has strong impact on the ecosystem and human health due to chronic exposure (1). In China, the amount of anthropogenic arsenic (As) is a big challenge as As emission is high in current industrialized scale (2). Other studies report that As exposure acts in a dose-effect manner in different tissues, such as the skin system (3). Toxicologic mechanisms have revealed that As-induced cytotoxicity may be involved in cellular oxidative stress, apoptosis, thiamine deficiency, and acetyl cholinesterase loss (4). In China, As-related skin lesions in patients have been linked to chronic exposure to As, resulting in poor skin health and quality of life (5). Other mechanical evidence shows that As-induced skin impairment may be involved in the activation of miR-155-5p, keratin 1, keratin 10, and keratin 6c in dermal samples (6). Additionally, occupational contact dermatitis is often observed in workers with prolonged exposure to As (7). Hence, there is an urgent need for effective procedures to reduce As-induced dermal toxicity. Vitamin A (VA), a well-known nutrient substance, is used extensively in medical treatment and health care due to its multi-functional features (8). Low-serum VA levels have been linked to inflammation in participants with retinol deficiency (9). VA deficiency has been reported to mediate some actions in the pathophysiology of atopic dermatitis *via* potentiating T-cell-related inflammation development (10). Meanwhile, primary clinical findings indicate that VA supplementation may relieve atopic dermatitis in children (11). There are reports that the physiological actions of VA are associated with the enhancement of skin immunity and maintenance of dermal microbiome (12). However, there are no reports regarding the anti-As-linked dermatitis activity and drug targets of VA treatment. As a drug development tool, network pharmacology is adequate for potential drug discovery in pharmacological target and mechanism in the treatment of clinical diseases (13, 14). Moreover, our group research findings based on network pharmacology approach and molecular docking analysis have been published, including the use of vitamin C against leukemia (15), sepsis (16), and calycosin for the treatment of meningitis (17) and cerebral ischemia/reperfusion injury (18). Therefore, in this study, we aimed to determine the therapeutic effects of VA against As-linked dermatitis and core drug targets *in silico* and to reveal the pharmacological pathways on the basis of network pharmacological assay and molecular docking technology.

## Materials and Methods

### Gene/Target Data Preparation

Vitamin A-related genes were obtained from the Comparative Toxicogenomics Database (19), the Swiss Target Prediction, and the PharmMapper databases. Some of the raw data were corrected *via* reviews (Swiss-Prot) and Human screening in the Uniprot database (20). Information regarding As-related dermatitis genes was obtained through the GeneCards and the Online Mendelian Inheritance in Man (OMIM) databases (21). All genes/targets were collected and submitted to “Venn diagram” setting in the R-language software to determine the connective targets in VA- and As-related dermatitis.

### Construction of Interactions in Selected Genes and Identification of Core Targets

All mutual genes identified were imported to STRING^1^ to establish the protein-protein interaction (PPI) network, with a confidence coefficient of 0.9. In addition, colorized nodes were denoted as target proteins, and interlaced edges were denoted as target gene-gene interactions (22). As reported previously (23), core targets were identified with degree algorithm value using the Network Analyzer setting in the Cytoscape v3.8.2 software.

### Enrichment Analyses of Gene Ontology and Kyoto Encyclopedia of Genes and Genomes

All core targets were submitted to the R-language plug-in, including “ClusterProfiler” and “GOplot,” for enrichment analysis and visualization. When enriched with *p*-value and *q*-value cutoff = 0.05, respective bubble, bar, and circle charts were created accordingly (24). In addition, the mutual integration networks indicating VA-target-function-pathway-dermatitis-related As were produced through Cytoscape (25).

### Molecular Docking Test of Core Target Proteins

Molecular docking imitation *in silico* was used to identify the binding affinity of the screening core target protein to dock with VA. The docking processes were conducted using the Chem Bio Office 2010 and the Autodock tools after some core target genes were screened down *via* a scored algorithm. The chemical structure of VA was obtained from PubChem,^2^ and the protein bioinformatics of the identified core targets was obtained using the Protein Data Bank database (26). Generally defined, root mean square deviation ≤ 4 Å is the threshold for docking conformation.

### Metabolic Pathway Enrichment Analysis

Core genes were uploaded to the MetaboAnalyst 5.0 database (27) to analyze the metabolic pathways. After matched information, the Metabolic pathways (integrated) database and the Hypergeometric Test enrichment algorithm were used for metabolic-pathway enrichment.

## Results

### Bioinformatics Mining in Candidate Targets

**FIGURE 1:**
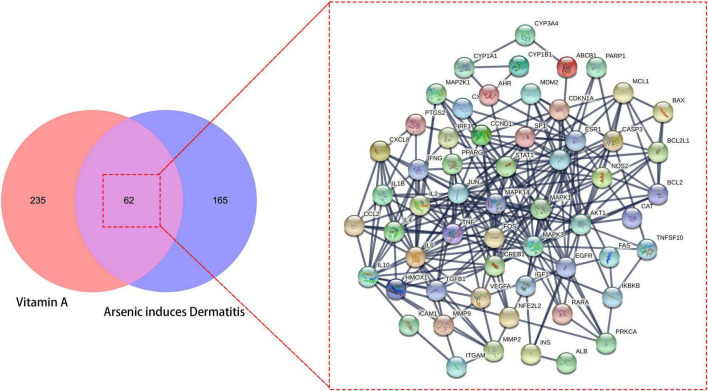
All candidates, mutual target genes in vitamin A (VA) drug repurposing for As-linked dermatitis in Venn diagram, as characterized in an interlaced network.

A total of 297 human genes related to VA were obtained from the online databases. We identified 227 common genes in As-linked dermatitis through data mining. With the Venn diagram, 62 overlapped genes between VA- and As-linked dermatitis were identified, and these intersection genes were further processed to produce the interlacement network (Figure 1).

### Ascertainment of Core Targets

**FIGURE 2:**
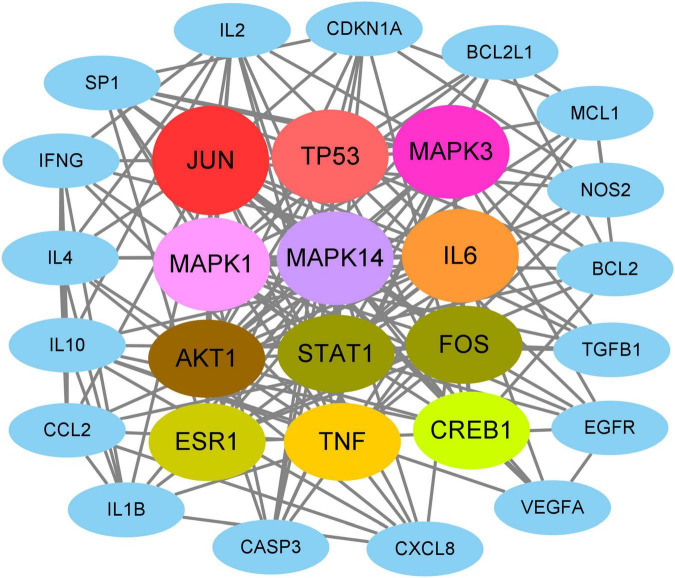
Identification of all core targets involved in VA against As-linked dermatitis.

On the basis of network topology algorithm, all core target genes in VA against As-linked dermatitis were reported, including *JUN*, *TP53*, *MAPK3*, *MAPK1*, *MAPK14*, *IL6*, *AKT1*, *STAT1*, *FOS*, *ESR1*, *TNF*, *CREB1*, *IL10*, *IL2*, *SP1*, *CASP3*, *CDKN1A*, *IL4*, *EGFR*, *IL1B*, *MCL1*, *BCL2*, *CXCL8*, *TGFB1*, *IFNG*, *BCL2L1*, *NOS2*, *CCL2*, and *VEGFA* (Figure 2). The scoring prioritization of these core targets was characterized through the coloration degree of nodes.

### Gene Ontology and Kyoto Encyclopedia of Genes and Genomes Enrichment Analysis Reports

**FIGURE 3:**
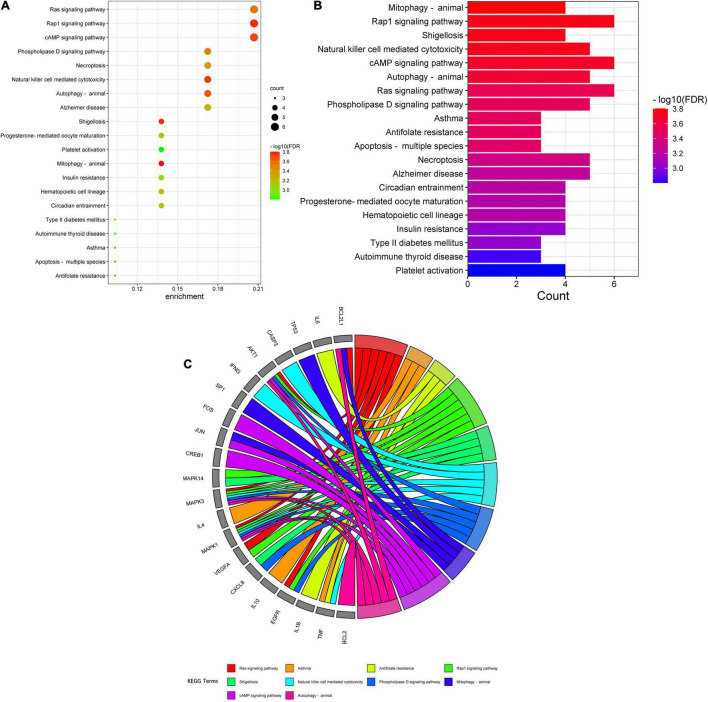
Gene ontology enrichment analysis findings in VA against As-linked dermatitis, as revealed in bubble chart **(A)**, bar chart **(B)**, circle chart **(C)**.

**FIGURE 4:**
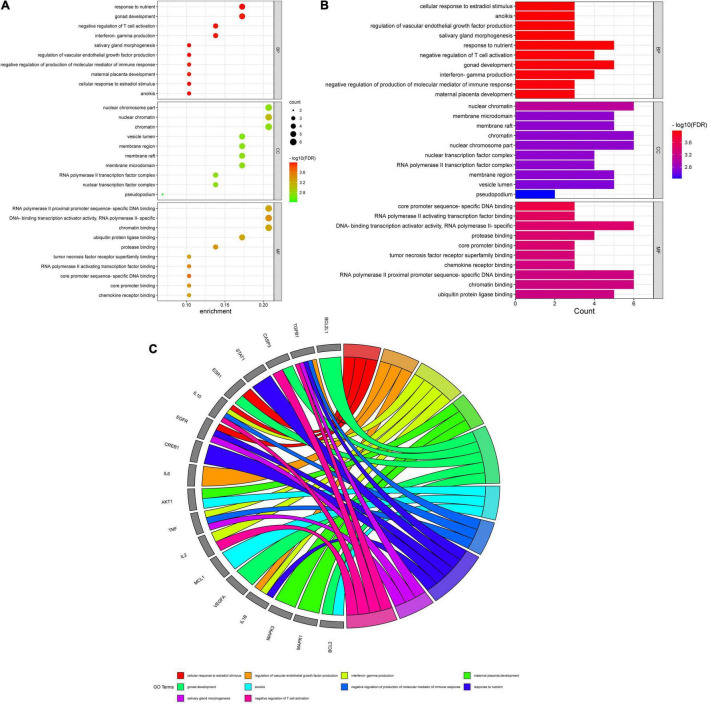
Kyoto Encyclopedia of Genes and Genomes enrichment assay data in VA against As-linked dermatitis, as uncovered in bubble **(A)**, bar **(B)**, and circle **(C)** charts.

**FIGURE 5:**
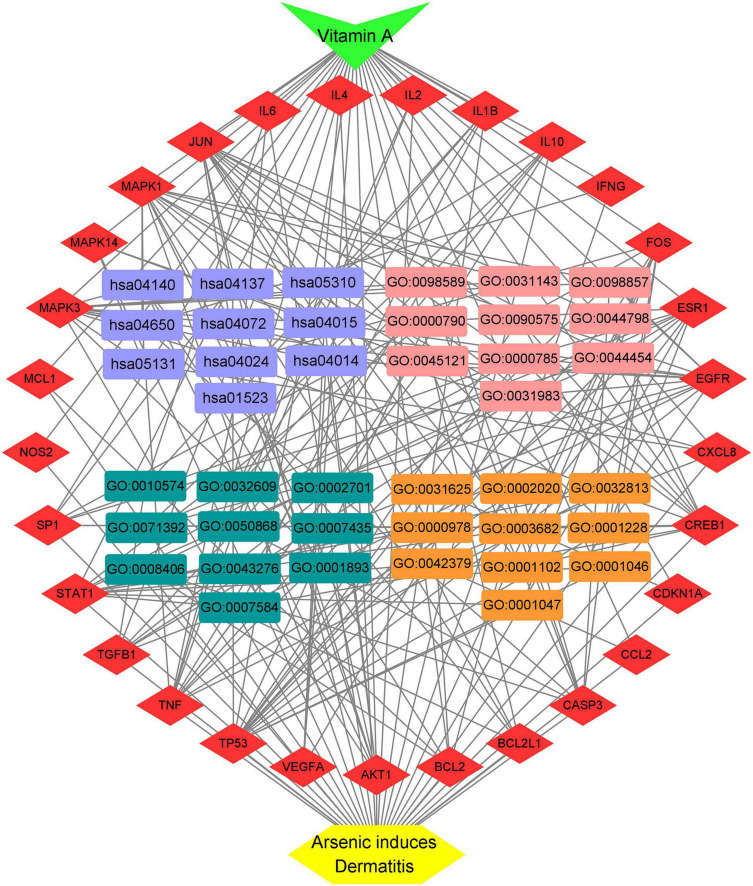
VA-targets-pathways-As-linked dermatitis network characterization.

A total of 151 Encyclopedia of Genes and Genomes (KEGG) molecular pathways enriched (*P* < 0.05) were identified as multiple key signaling pathways, comprising mitophagy-animal, Rap1 signaling pathway, shigellosis, natural killer-cell mediated cytotoxicity, cAMP signaling pathway, autophagy-animal, Ras signaling pathway, phospholipase D signaling pathway, antifolate resistance, asthma, apoptosis-multiple species, necroptosis, Alzheimer’s disease, circadian entrainment, hematopoietic cell lineage, progesterone-mediated oocyte maturation, insulin resistance, type II diabetes mellitus, autoimmune thyroid disease, and platelet activation. The bubble charts of KEGG pathway enrichments are shown in Figure 3A, the bar chart in Figure 3B, and the circle chart in Figure 3C. Gene Ontology (GO) enrichment analysis further revealed multiple VA-anti-dermatitis functions related to As, including biological processes, cellular components, and molecular functions. The output data are demonstrated in bubble chart (Figure 4A), histogram (Figure 4B), and circle chart (Figure 4C). All genes and other enrichment data were merged for the establishment of the VA-target-pathway-As-linked dermatitis network (Figure 5).

### Molecular Docking Imitation Data

**FIGURE 6:**
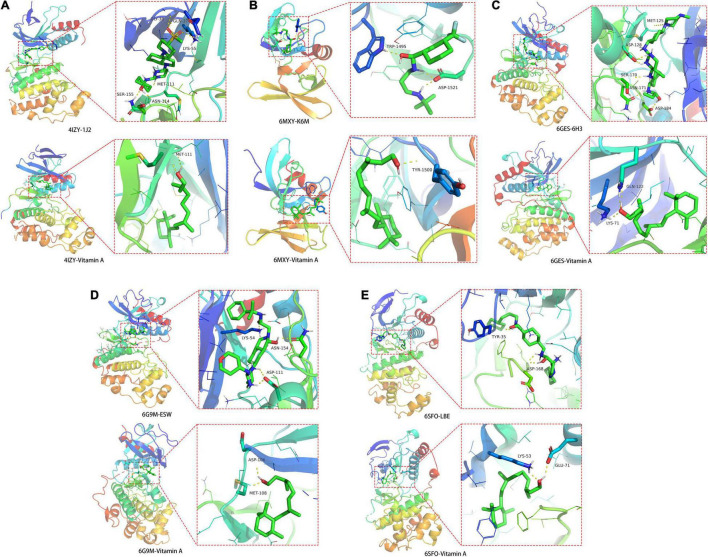
Molecular docking details of **(A)** JUN-4IZY; **(B)** TP53-6MXY; **(C)** MAPK3-6GES; **(D)** MAPK1-6G9M; and **(E)** MAPK14-6SFO in VA action with core target proteins.

The crystal structures for JUN (PDB ID: 4IZY), TP53 (PDB ID: 6MXY), MAPK3 (PDB ID: 6GES), MAPK1 (PDB ID: 6G9M), and MAPK14 (PDB ID: 6SFO) were screened out for docking determination. In Figure 6A, the original ligand 1J2 chemically bonded with 4IZY crystal protein through GLY-38, GLN-37, LYS-55, MET-111, SER-155, and ASN-114 amino acid residues, and the free docking energy was –7.18 kcal/mol. VA docked well with MET-111 with –5.70 kcal/mol free docking energy. In Figure 6B, the original ligand K6M chemically bonded with 6MXY crystal protein though TRP-1495 and ASP-1521 amino acid residues, and the free docking energy was –6.72 kcal/mol. VA docked well with TYR-1500 with –5.52 kcal/mol free docking energy. In Figure 6C, the original ligand 6H3 chemically bonded with 6GES crystal protein though MET-125, ASP-128, ASN-171, SER-170, and ASP-184 amino acid residues, and the free docking energy was –9.83 kcal/mol. VA docked well with GLN-122 and LYS-71 with –5.72 kcal/mol free docking energy. In Figure 6D, the original ligand ESW chemically bonded with 6G9M crystal protein though LYS-54, ASN-154, and ASP-111 amino acid residues with a free docking energy of –7.71 kcal/mol. VA docked well with ASP-106 and MET-108 with –5.47 kcal/mol free docking energy. In Figure 6E, the original ligand LBE chemically bonded with 6SFO crystal protein though TYR-35 and ASP-168 amino acid residues, and the free docking energy was –10.28 kcal/mol. VA docked well with LYS-53 and GLU-71 with –7.9 kcal/mol free docking energy.

### Metabolic Pathway Enrichment Report

**FIGURE 7:**
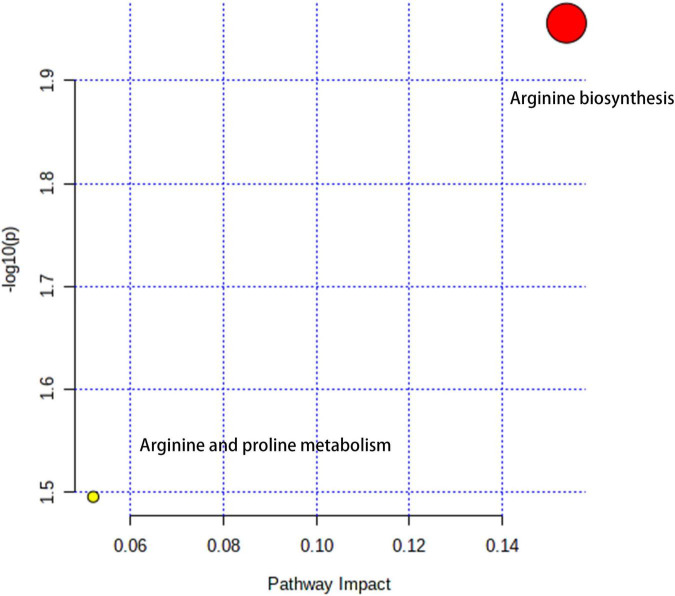
Enrichment reports of metabolic pathways in core targets.

The metabolic pathways were enriched with Arginine biosynthesis and Arginine and proline metabolism, mainly characterized by the NOS2 signaling pathway (Figure 7).

## Discussion

Repurposing an existing drug can be achieved through bioinformatics analysis, such as network pharmacology approach (14). Identifying the existing VA drug with multiple targets may be potentially useful against some clinical diseases, such as COVID-19 (28). In this study, a bioinformatics methodology for complete identification of repurposing VA is used to predict the pharmacological mechanisms for potential treatment of dermatitis associated with As, a cytotoxic metallic element. Integration analysis of VA-target-pathway-dermatitis related to As findings and complete identification of PPI networks are essential for further biological structural docking. With network pharmacology determination, we recognized 62 candidate targets and 29 core drug targets in VA for treating As-related dermatitis. As shown in all core targets, JUN, TP53, MAPK3, MAPK1, MAPK14, IL6, AKT1, STAT1, FOS, ESR1, TNF, CREB1, IL10, IL2, SP1, CASP3, CDKN1A, IL4, EGFR, IL1B, MCL1, BCL2, CXCL8, TGFB1, IFNG, BCL2L1, NOS2, CCL2, and VEGFA were identified to be virtually associated with As-related dermatitis. Cytokines have been proven to possess pro-inflammation and immunodepression actions. Cytokines have developed promptly, leading to the induction of dermatitis (29). Previous evidence has suggested that As impacts the production of inflammatory cytokines in the skin, causing allergic dermatitis (30). VA is well evidenced as an anti-inflammatory micronutrient as it has a key role in heightening immune function (31). Thus, the anti-inflammatory action of VA is employed in pharmacologically treating As-related dermatitis. Low concentration of arsenic trioxide may induce human keratinocyte growth but concomitantly affects the DNA transcription function (32). Cyclins are the potential biomarkers of mild atopic dermatitis, characterized by molecular signatures in dermatitis cases (33). Additionally, it is important to note the modulation of the cyclin pathway in the treatment of As-related dermatitis with VA. As shown in further molecular docking imitation, *in silico* findings suggested that VA structurally docked well with some core target proteins, including JUN, TP53, MAPK3, MAPK1, and MAPK14. JUN can directly interact with specific target DNA sequences to regulate gene expression (34). JUN is one of the most important components of the activated protein-1 (AP-1) transcription factor complex, in which the increase in AP-1 activity can promote inflammation (35). In addition, c-Jun/AP-1 may serve as a potential pharmacological target in psoriatic skin inflammation by regulating immune responses (36). *TP53* is a well-known anti-apoptotic gene. In the event of functional loss, TP53-induced systemic inflammation occurs and evolves over time (37). Clinical observations have revealed abnormal p53 immunoreactive patterns in human skin lesions (38). MAPKs, such as MAPK3, MAPK1, and MAPK14, are signal regulators that are responsible for cell proliferation, apoptosis, and survival through the regulation of inflammatory cytokines and growth factors (39). In particular, MAPK3 is activated through inflammatory cytokines and extracellular stimulation (40). Thereby, immediate modulation of MAPK activation may be another molecular mechanism for VA action in the treatment of As-related dermatitis. In this study, we proposed a computational methodology for the prediction of the VA compound for treatment against As-related dermatitis, detailed with core targets and pharmacological mechanisms. One of the limitations of our study is that our findings were mostly collected from publicly available databases, and some of the correlative data may be incomplete. As potential limitations in this study, preclinical study needs to be determined for *in vitro* therapeutic effectiveness and adverse actions *in vivo* prior to future clinical validation.

## Conclusion

This study provides a potent, integrative pharmacological approach for the effective identification of repurposing VA as a potential therapy for As-related dermatitis. Using this bioinformatics methodology, we can reduce the translational period in clinical trials, resulting in rapid therapy strategy for the use of VA in As-related dermatitis.

## Data Availability Statement

The original contributions presented in the study are included in the article/Supplementary Material, further inquiries can be directed to the corresponding author/s.

## Author Contributions

CG and BY contributed to the conception, design of the manuscript, drafted the manuscript, and revised the manuscript. QQ, LQ, RX, and SP contributed to the acquisition, analysis, and interpretation of data in the manuscript. All authors agreed to be accountable for all aspects of work ensuring integrity and accuracy.

## Conflict of Interest

The authors declare that the research was conducted in the absence of any commercial or financial relationships that could be construed as a potential conflict of interest.

## Publisher’s Note

All claims expressed in this article are solely those of the authors and do not necessarily represent those of their affiliated organizations, or those of the publisher, the editors and the reviewers. Any product that may be evaluated in this article, or claim that may be made by its manufacturer, is not guaranteed or endorsed by the publisher.

## Footnotes

1
https://string-db.org/cgi/network.pl

2
https://pubchem.ncbi.nlm.nih.gov
